# Effects of Stochastic Noises on Limit-Cycle Oscillations and Power Losses in Fusion Plasmas and Information Geometry

**DOI:** 10.3390/e25040664

**Published:** 2023-04-15

**Authors:** Rainer Hollerbach, Eun-jin Kim

**Affiliations:** 1 Department of Applied Mathematics, University of Leeds, Leeds LS2 9JT, UK; r.hollerbach@leeds.ac.uk; 2 Centre for Fluid and Complex Systems, Coventry University, Priory St, Coventry CV1 5FB, UK

**Keywords:** entropy, entropy-production, mutual information, information flow, information geometry, information length, information rate, oscillations, stochastic noise, ELMs, plasmas

## Abstract

We investigate the effects of different stochastic noises on the dynamics of the edge-localised modes (ELMs) in magnetically confined fusion plasmas by using a time-dependent PDF method, path-dependent information geometry (information rate, information length), and entropy-related measures (entropy production, mutual information). The oscillation quenching occurs due to either stochastic particle or magnetic perturbations, although particle perturbation is more effective in this amplitude diminishment compared with magnetic perturbations. On the other hand, magnetic perturbations are more effective at altering the oscillation period; the stochastic noise acts to increase the frequency of explosive oscillations (large ELMs) while decreasing the frequency of more regular oscillations (small ELMs). These stochastic noises significantly reduce power and energy losses caused by ELMs and play a key role in reproducing the observed experimental scaling relation of the ELM power loss with the input power. Furthermore, the maximum power loss is closely linked to the maximum entropy production rate, involving irreversible energy dissipation in non-equilibrium. Notably, over one ELM cycle, the information rate appears to keep almost a constant value, indicative of a geodesic. The information rate is also shown to be useful for characterising the statistical properties of ELMs, such as distinguishing between explosive and regular oscillations and the regulation between the pressure gradient and magnetic fluctuations.

## 1. Introduction

Stochastic noises have interesting effects on nonlinear dynamical systems, including the change in stability, noise-induced stochastic resonance [1,2,3,4], pattern formation [5], and noise-delayed extinction [6]. In particular, their dual role of stabilising an unstable equilibrium point and destabilising a stable equilibrium point can be seemingly counter-intuitive. Furthermore, given the ubiquity of oscillations in diverse fields (e.g., physics, chemistry, biology, fluid/plasmas systems, ecological systems, population dynamics, environment dynamics, etc.), the so-called oscillation quenching receives considerable attention, whereby oscillations are suppressed through oscillation death or amplitude death [4,5,7,8]. In the context of a coupled dynamical system, it can be caused by a subtle change in the coupling, such as time-delay, a parameter mismatch, etc. [9,10].

Some oscillations occurring in nature or a laboratory can be quite explosive, with the potential to cause undesirable damage to a system. An important example is quasi-periodic, limit-cycle oscillations in magnetically confined plasmas [11], such as edge-localised modes (ELMs) [12,13,14,15,16,17,18,19,20,21] and sawtooth oscillations [11]. Specifically, ELMs occur due to instabilities of pressure/current gradient at edge plasmas for a sufficiently high input power in the high-confinement mode (H-mode) regime (see, e.g., [22,23,24,25] and references therein) and take the form of sudden, quasi-periodic oscillations/bursts or more regular oscillations. The former, bursty, large-amplitude (Type I) ELMs can damage the fusion device walls, and thus have stimulated various research efforts to suppress or mitigate such ELMs. The two main mechanisms invoke the injection of particle or magnetic field perturbations externally (e.g., resonant magnetic perturbations or a pellet injection in DIII-D, JET, ASDEX-U, EAST, KSTAR tokamaks [16,17,20]). In particular, the effect of magnetic perturbation requires attention even at a very small kinetic level [26,27].

Some of the interesting experimental observations are that the ELM suppression and mitigation depend on electron pedestal collisionality, density, edge safety factor, plasma boundary shape, as well as the characteristics of the magnetic perturbation, such as the amplitude, toroidal and poloidal mode numbers, and the amplitude/frequency of (particle) pallets [18,19]. For resonant magnetic perturbations, a stochastic layer can form on the edge, promoting rapid radial transport. However, the direct impact of stochastic magnetic fields on ELM suppression/mitigation is not well understood, given its difficulty in experimental measurements. It is thus worth studying the effect of stochastic magnetic perturbation on ELMs in simple models to gain a key understanding.

The main focus of this paper is to provide detailed study of the effects of stochastic particles and magnetic perturbations on ELMs. In our previous work [28], we proposed a stochastic ELM model based on a minimal deterministic ODE model of ELM dynamics in [12], which evolves the pressure gradient, magnetic fluctuation amplitude together with the ion radial-force balance relation where the electric fields are driven by the pressure gradient and the poloidal velocity. By varying the strength of the stochastic particle perturbation for a fixed small stochastic magnetic perturbation, we demonstrated their non-trivial effects on ELM dynamics, e.g., in altering the amplitude and period of oscillations.

In this paper, we extend the analysis in [28] and perform a systematic study by varying the strength of both stochastic external particle and magnetic perturbations to elucidate their effects on the characteristics of ELM oscillations and associated power/energy loss. We will explore a smaller value of the stochastic noise than previously considered in [28] and also scan over different strengths of these stochastic noises. In particular, we aim to address the following main questions:

- How are ELMs affected by stochastic particle and magnetic perturbation?
- Which noise is more effective in reducing the maximum power loss due to ELMs?
- How far from equilibrium is the system driven due to ELMs in the presence of the stochastic noises?
- How are power loss and energy loss due to ELMs affected by the stochastic noises and input power?
- How are power loss and energy loss due to ELMs captured by different statistical measures?
- What are robust diagnostics to identify explosive versus regular small ELMs?

In order to answer these questions, we calculate time-dependent probability density, power loss, information geometry diagnostics [29,30], and entropy-related measures [31] for different cases. Specifically, our path-dependent information geometry [25,28,32,33] allows us to quantify the changes in time-dependent PDFs along the evolution path. The entropy production rate, which measures the rate of irreversible energy dissipation in a non-equilibrium system [32,34,35,36], will be explored for possible links between the maximum power loss, entropy production, and information geometric diagnostics.

The remainder of this paper is organised as follows. We present our stochastic ELM model in Section 2 and a brief recap of our information geometry diagnostics and other entropy diagnostics in Section 3 to make the paper self-contained. Section 4 and Section 5 provide our numerical methods and results, respectively. We conclude in Section 6. Appendix A presents the results that are not included in the main text of the paper.

## 2. Model

Our stochastic model of ELMs evolves two variables, *x* and *y*, which are related to the dimensionless pressure gradient *p* and the square-root of magnetic fluctuations $E_{M}$, respectively, as x=p and $y=\sqrt{E_{M}}$, as follows:

(1) $$\begin{array}{ccc} \frac{dx}{dt} & = & \Phi -\tilde{D}\left(x\right)x-xy^{2}+\xi =f+\xi ,\;\; \end{array}$$

(2) $$\begin{array}{ccc} \frac{dy}{dt} & = & \frac{1}{2}\lambda \left(x-1\right)y+\eta =g+\eta , \end{array}$$

(3) $$\begin{array}{ccc} \tilde{D} & = & d_{0}+d\left(x-c^{2}x^{4}\right)\Theta \left(\tilde{P}-x\right). \end{array}$$

Here, Θ(x) is the Heaviside function with Θ(x)=1 for x≥0 and Θ(x)=0 for x<0; $\tilde{P}$ is the critical pressure gradient *x* above which turbulence is completely suppressed with no turbulent transport $d\left(x-c^{2}x^{4}\right)\Theta \left(\tilde{P}-x\right)\to 0$, where $c\equiv {\tilde{P}}^{-3/2}$. λ and $d_{0}\ll d$ are non-negative constants. Φ is an external particle source term.

We note that Equations (1)–(3) are based on the following assumptions:

- The temperature is constant so that Φ (particle sources) plays a role as the control parameter of the energy flux (input power);
- The input power $P_{in}$ is much greater than the critical power-threshold $P_{cr}$ so that the electric field is mainly driven by the pressure gradient (diamagnetic velocity);
- There is no ELMy free H-mode gap;
- Time is nondimensionalised by ${\left[\left(c_{s}/\rho_{s}\right)k\rho_{s}\left(\Delta_{c}^{4}/\rho_{s}^{2}L_{p}^{2}\right)\right]}^{-1}$, where $c_{s}=\sqrt{T_{e}/m_{i}}$ is the ion sound speed, $\rho_{s}=c_{s}/\omega_{ci}$, $\omega_{ci}$ is the ion cyclotron frequency, and *k* and $\Delta_{c}$ are the poloidal wave number and radial correlation length of the turbulence (see [12]).

We note that the condition $P_{in}\gg P_{cr}$ above corresponds to $P_{in}/P_{cr}\gg \left(R\rho_{s}L_{p}^{2}\right){\left(\rho_{s}/L_{p}\right)}^{1/3}$ when the pressure gradient (the diamagnetic velocity) becomes sufficiently large to dominate the contribution from the poloidal velocity. Here, *R*, $\rho_{s}=c_{s}/\omega_{ci}$, $c_{s}=\sqrt{T_{e}/m_{i}}$, $\omega_{ci}$, $L_{p}$, and *a* represent the major radius, the ion Larmor radius calculated from the ion sound speed, the sound speed, the ion cyclotron frequency, the length-scale of the pressure gradient, and the minor radius, and ion sound speed, respectively [12].

In Equations (1)–(3), ξ and η are two independent Gaussian noises of strength $Q_{x}$ and $Q_{y}$, with the following statistical properties

(4) $$\begin{array}{ccc} \langle \xi \left(t\right)\xi \left(t^{\prime }\right)\rangle =2Q_{x}\delta \left(t-t^{\prime }\right),\;\; & & \langle \eta \left(t\right)\eta \left(t^{\prime }\right)\rangle =2Q_{y}\delta \left(t-t^{\prime }\right),\\ \langle \xi \left(t\right)\eta \left(t^{\prime }\right)\rangle =0,\;\;\;\; & & \langle \xi \rangle =\langle \eta \rangle =0, \end{array}$$

where $\delta (t-t^{\prime })$ represents a short memory time of ξ and η in comparison with any other characteristic time scales (e.g., ELM period) in the system. ξ and η in Equations (1) and (2) are included to capture any external stochastic perturbation or stochastic events/transport in the systems, such as fluctuating energy flux of unresolved scales, the outward energy flux at the edge (e.g., [37,38]), pellet pacing [16], mini-avalanches [25], stochastic magnetic fields, kinetic instabilities (due to the runaway electrons) [27], or external magnetic coils, etc. Given the uncertainty in the values of $Q_{x}$ and $Q_{y}$, our interest in this paper is the trend in the effects of $Q_{x}$ and $Q_{y}$ on ELM dynamics.

### The Fokker–Planck Equation PDF

To ensure the accurate calculation of time-dependent PDFs and information diagnostics, we will use the Fokker–Planck method [39] instead of stochastic simulations of Equations (1)–(3) [40]. Corresponding to the Langevin model in Equations (1)–(3), the Fokker–Planck equation [39] for the joint PDF p(x,y,t) is given by

(5) $$\begin{array}{ccc} \frac{\partial p}{\partial t} & = & -\frac{\partial }{\partial x}\left(f\;p\right)-\frac{\partial }{\partial y}\left(g\;p\right)+Q_{x}\frac{\partial^{2}p}{\partial x^{2}}+Q_{y}\frac{\partial^{2}p}{\partial y^{2}}=-\partial_{x}J_{x}-\partial_{y}J_{y}. \end{array}$$

Here,

(6) $$\begin{array}{ccc} & & f\equiv \Phi -\tilde{D}\left(x\right)x-xy^{2},\;g\equiv \frac{\lambda }{2}\left(x-1\right)y, \end{array}$$

(7) $$\begin{array}{ccc} & & J_{x}=fp-Q_{x}\partial_{x}p,\;J_{y}=gp-Q_{y}\partial_{y}p, \end{array}$$

where $J_{x}$ and $J_{y}$ are the probability currents of *x* and *y*.

The equations for marginal PDFs p(x,t)=∫dyp(x,y,t) and p(y,t)=∫dxp(x,y,t) are obtained by integrating Equation (5) over *y* or *x*, respectively

(8) $$\begin{array}{ccc} \frac{\partial p(x,t)}{\partial t} & = & -\frac{\partial }{\partial x}\int dy\;\left(f\;p\right)+Q_{x}\frac{\partial^{2}p}{\partial x^{2}}, \end{array}$$

(9) $$\begin{array}{ccc} \frac{\partial p(y,t)}{\partial t} & = & -\frac{\partial }{\partial y}\int dx\;\left(g\;p\right)+Q_{y}\frac{\partial^{2}p}{\partial y^{2}}. \end{array}$$

Here, we used the boundary conditions $J_{x}\left(x\to \pm \infty ,y,t\right)=0$ (p(x→±∞,y,t)=0) and $J_{y}\left(x,y\to \pm \infty ,t\right)=0$ (p(x,y→±∞,t)=0).

## 3. Information Geometry, Entropy Production, and Power Loss

### 3.1. Information Rate, Length

We quantify the temporal change in PDF along its trajectory using the information rate and length. Specifically, for a one-variable system with a PDF p(x,t), they are given by [25,32]

(10) $$\begin{array}{ccc} \Gamma^{2}\left(t\right) & = & \int dx\frac{1}{p(x,t)}{\left[\frac{\partial p(x,t)}{\partial t}\right]}^{2},\\ L(t) & = & \int_{0}^{t}dt_{1}\Gamma \left(t_{1}\right).\; \end{array}$$

We recall that in Equation (10), Γ, has units of inverse time, which quantifies the rate at which a PDF changes in time; the dimensionless L(t) quantifies the total cumulative change in a PDF—the total number of statistically different states that *x* passes through between time 0 and *t*. Further, we recall that for the Gaussian PDFs, L(t) represents the cumulative change in p(x,t) measured in units of standard deviation. Given its path-dependent property of L (being calculated along the PDF trajectory), it is useful for quantifying dynamical hysteresis involved in phase transitions such as the L-H transition and characterising nonlinear dynamical systems (e.g., attractors, stability/instability) [32].

For the two variables *x* and *y*, we calculate Γ and L from the joint PDF p(x,y,t) and $\Gamma_{x}$, $\Gamma_{y}$, $L_{x}$, and $L_{y}$ from the marginal PDFs p(x,t) and p(y,t) as

(11) $$\begin{array}{ccc} & & L_{}\left(t\right)=\int_{0}^{t}dt_{1}\Gamma \left(t_{1}\right), \end{array}$$

(12) $$\begin{array}{ccc} & & \Gamma^{2}\left(t\right)=\int dxdy\frac{1}{p(x,y,t)}{\left[\frac{\partial p(x,y,t)}{\partial t}\right]}^{2},\;\;\; \end{array}$$

(13) $$\begin{array}{ccc} & & L_{x}\left(t\right)=\int_{0}^{t}dt_{1}\Gamma_{x}\left(t_{1}\right), \end{array}$$

(14) $$\begin{array}{ccc} & & L_{y}\left(t\right)=\int_{0}^{t}dt_{1}\Gamma_{y}\left(t_{1}\right), \end{array}$$

(15) $$\begin{array}{ccc} & & \Gamma_{x}^{2}\left(t\right)=\int dx\frac{1}{p(x,t)}{\left[\frac{\partial p(x,t)}{\partial t}\right]}^{2}, \end{array}$$

(16) $$\begin{array}{ccc} & & \Gamma_{y}^{2}\left(t\right)=\int dy\frac{1}{p(y,t)}{\left[\frac{\partial p(y,t)}{\partial t}\right]}^{2}. \end{array}$$

Utilising the invariance of Equations (13)–(16) under a (time-independent) change in variables, we will examine how *x* and *y* are correlated and self-regulated (e.g., [25,33]) in explosive versus small ELMs in Section 5.4. Note that, in our model, *x* and *y* are dependent on $\Gamma^{2}\neq \Gamma_{x}^{2}+\Gamma_{y}^{2}$.

### 3.2. Entropy, Entropy Production, and Entropy Flow

We recall differential entropies $S_{x}$, $S_{y}$, and *S* based on the marginal PDFs p(x,t) and p(y,t) and the joint PDF p(x,y,t), respectively, and mutual entropy *I* as follows [31,32]:

(17) $$\begin{array}{ccc} S_{x} & = & -\int dx\;p(x,t)\ln\left(p(x,t)\right), \end{array}$$

(18) $$\begin{array}{ccc} S_{y} & = & -\int dy\;p(y,t)\ln\left(p(y,t)\right), \end{array}$$

(19) $$\begin{array}{ccc} S & = & -\int dxdy\;p(x,y,t)\ln\left(p(x,y,t)\right), \end{array}$$

(20) $$\begin{array}{ccc} I & = & S_{x}+S_{y}-S=\int dxdy\;p\left(x,y,t\right)\ln\frac{p(x,y,t)}{p(x,t)p(y,t)}. \end{array}$$

As a measure of irreversibility, we consider the total entropy production

(21) $$\begin{array}{c} {\dot{S}}_{T}=\dot{S}+{\dot{S}}_{m}, \end{array}$$

where ${\dot{S}}_{m}$ is the entropy flow rate (entropy flux to the environment at temperature $Q_{x}$ and $Q_{y}$), and the two terms are defined by

(22) $$\begin{array}{c} {\dot{S}}_{T}=\int dxdy\left[\left(\frac{1}{Q_{x}p}J_{x}^{2}+\frac{1}{Q_{y}p}J_{y}^{2}\right)\right],\;\;{\dot{S}}_{m}=\int dxdy\left[\left(\frac{1}{Q_{x}}J_{x}f+\frac{1}{Q_{y}}J_{y}g\right)\right]. \end{array}$$

It is important to note that ${\dot{S}}_{T}$ and ${\dot{S}}_{m}$ in Equation (22) are calculated from a joint PDF, and can be shown to take the following form

(23) $$\begin{array}{ccc} {\dot{S}}_{T} & = & {\dot{S}}_{Tx}+{\dot{S}}_{Ty}, \end{array}$$

(24) $$\begin{array}{ccc} {\dot{S}}_{m} & = & {\dot{S}}_{mx}+{\dot{S}}_{my}, \end{array}$$

where

(25) $$\begin{array}{c} {\dot{S}}_{Tx}=\int dxdy\frac{1}{Q_{x}p}J_{x}^{2},\;\;\;{\dot{S}}_{Ty}=\int dxdy\frac{1}{Q_{y}p}J_{y}^{2}, \end{array}$$

(26) $$\begin{array}{c} {\dot{S}}_{mx}=\int dxdy\frac{1}{Q_{x}}J_{x}f,\;\;\;{\dot{S}}_{my}=\int dxdy\frac{1}{Q_{y}}J_{y}g. \end{array}$$

${\dot{S}}_{Tx}$ and ${\dot{S}}_{Ty}$ (${\dot{S}}_{mx}$ and ${\dot{S}}_{my}$) represent the entropy production rates in *x* and *y* (entropy *x* and *y* to their heat baths), respectively.

For independent *x* and *y* we have that ${\dot{S}}_{Tx}={\dot{S}}_{x}+{\dot{S}}_{mx}$ and ${\dot{S}}_{Ty}={\dot{S}}_{y}+{\dot{S}}_{my}$, but in general, these do not hold due to the interaction between *x* and *y*. These relations, therefore, need to be generalised as follows [28]:

(27) $$\begin{array}{ccc} {\dot{S}}_{Tx}={\dot{S}}_{x}+{\dot{S}}_{mx}-T_{y\to x}, & & {\dot{S}}_{Ty}={\dot{S}}_{y}+{\dot{S}}_{my}-T_{x\to y}. \end{array}$$

Here, $T_{x\to y}$ and $T_{y\to x}$ are the information rate from *x* to *y* and *y* to *x*, respectively, given by

(28) $$\begin{array}{ccc} T_{y\to x} & = & \partial_{\tau }{I\left(x\left(t+\tau \right),y\left(t\right)\right)|}_{\tau \to 0}=-\int dxdy\;J_{x}\left(x,y,t\right)\partial_{x}\ln\left[\frac{p(x,t)}{p(x,y,t)}\right], \end{array}$$

(29) $$\begin{array}{ccc} T_{x\to y} & = & \partial_{\tau }{I\left(x\left(t\right),y\left(t+\tau \right)\right)|}_{\tau \to 0}=-\int dxdy\;J_{y}\left(x,y,t\right)\partial_{y}\ln\left[\frac{p(y,t)}{p(x,y,t)}\right], \end{array}$$

where *I* is the mutual information in Equation (20). It is emphasised that the rate at which the mutual information changes in time is the sum of $T_{x\to y}$ and $T_{y\to x}$ (see [28]):

(30) $$\begin{array}{ccc} \frac{dI}{dt} & = & T_{y\to x}+T_{x\to y}. \end{array}$$

### 3.3. Power Loss

One of the useful measures in fusion plasmas is the power loss $P_{L}$ representing how much power is lost through turbulent transport

(31) $$P_{L}=\Phi -\langle \frac{dx}{dt}\rangle =\langle \tilde{D}\left(x\right)x+xy^{2}\rangle .$$

To be able to quantify how explosive the power loss is compared to the input power, we define a normalised power loss as

(32) $$\bar{P_{L}}=\frac{P_{L}}{\Phi },$$

and use it to compare different cases in examining the effect of stochastic noises in Section 5.

## 4. Numerical Experiments

We numerically solve the Fokker–Planck Equations (5)–(7) by discretising *x* and *y* using second-order finite differences [28] with the resolution $∼10^{-3}$ in *x* and *y*. For time-stepping, we use the second-order Runge–Kutta with time steps as small as $2\times 10^{-5}$. We look for the symmetric solutions p(x,y,t)=p(x,−y,t) in *y* on physical basis and solve Equations (5)–(7) in a 2D-box in $x=[x_{min},x_{max}]$ and $y=[0,y_{max}]$ with the boundary conditions $p\left(x_{min},y,t\right)=p\left(x_{max},y,t\right)=p\left(x,y_{max},t\right)=0$, and $\frac{\partial p}{\partial y}=0$ at y=0. $x_{min}$, $x_{max}$, and $y_{max}$ are chosen to ensure that the solution becomes sufficiently small at the boundaries of the 2D-box, in particular, the total probability ∫∫p(x,y,t)dxdy=1 to within $10^{-4}$.

Since the purpose of this paper is to investigate the effect of stochastic noises $Q_{x}$ and $Q_{y}$ on oscillations at different values of Φ/d, we fix an initial condition to be a narrow Gaussian PDF with the mean values 〈x(0)〉=1.2 and 〈y(0)〉=0.2 and standard deviations $\sigma_{x}\left(0\right)=\sigma_{y}\left(0\right)=0.04$ while keeping the parameters $d_{0}=10^{-3}$, d=0.1, $\tilde{P}=1.05$, and λ=5. We consider the four different values Φ/d=0.6,0.8,1.0,1.2. Recall that in the deterministic model, as Φ/d is increased from Φ/d=0.4 to 1.2, ELMs gradually change from explosive events (giant ELMs) to more sinusoidal (grassy, small ELMs) with a shorter oscillation period. For each Φ/d, we vary the values of $Q_{x},Q_{y}=3\times 10^{-6},10^{-5},3\times 10^{-5},10^{-4},3\times 10^{-4},10^{-3}$.

## 5. Results

The previous work [28] fixed $Q_{y}$, and explored the effects of $Q_{x}$ and showed that random trajectories due to stochastic noise-induced phase-mixing where phase information is lost over time. Consequently, in the long time limit, p(x,y,t) will completely forget the phase information as well as the initial conditions and will reach a stationary PDF regardless of the initial conditions. In other words, in the stationary state, oscillations are completely suppressed with equal probability of all different phases. Furthermore, there was some indication of shortening the period of oscillation for a more explosive oscillation. In the following, we examine the results for different values of $Q_{x}$ and $Q_{y}$.

### 5.1. ODE Solution

**Figure 1:**
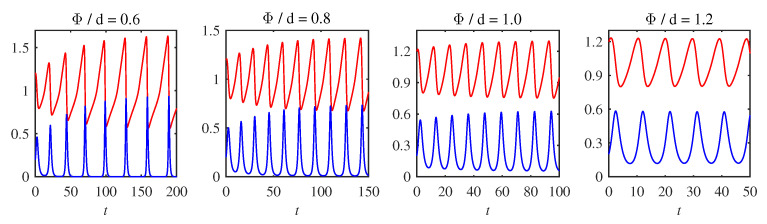
Deterministic solutions of Equations (1)–(3), with ξ=η=0 and initial conditions x=1.2 and y=0.2, $d_{0}=10^{-3}$, d=0.1, $\tilde{P}=1.05$, λ=5, and Φ/d=0.6,0.8,1.0,1.2 as indicated above each panel. Red denotes x=P, blue $y=\sqrt{E_{M}}$.

**Figure 2:**
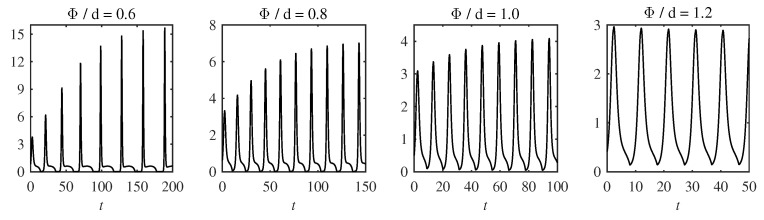
Normalised power loss against time for the deterministic solutions in Figure 1 for Φ/d=0.6, 0.8, 1.0, 1.2.

We start by looking at the ODE solution to appreciate the difference between the four cases Φ/d=0.6,0.8,1.0,1.2. Figure 1 shows the time-evolution of x=P and $y=\sqrt{E_{M}}$ in Equations (1)–(3) in the absence of noise ξ=η=0. It can easily be seen that the period of ELMs becomes smaller for larger Φ. For the smallest Φ/d=0.6, *x* (in red) slowly rises before suddenly collapsing back to a small value. This is triggered by the onset of the burst (spike) of *y* (in blue) due to the instability of the L-mode solution (x<1,y=0). Between the bursts, *y* spends a long time around the unstable *L*-mode solution (x<1,y=0). We also see that the oscillations for larger Φ/d become more regular/sinusoidal and more symmetric. Figure 2 shows the normalised power loss ${\bar{P}}_{L}$ defined in Equation (32) for the deterministic cases shown in Figure 1.

### 5.2. Mean and Standard Deviation

**Figure 3:**
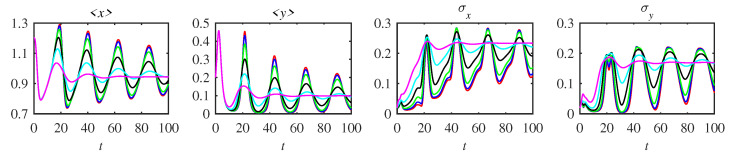
Φ/d=0.6: Scan over $Q_{x}=3\times 10^{-6},10^{-5},3\times 10^{-5},10^{-4},3\times 10^{-4},10^{-3}$ for $Q_{y}=10^{-5}$.

**Figure 4:**
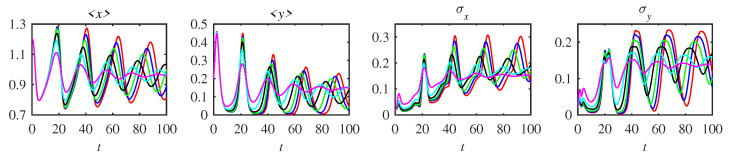
Φ/d=0.6: Scan over $Q_{y}=3\times 10^{-6},10^{-5},3\times 10^{-5},10^{-4},3\times 10^{-4},10^{-3}$ for $Q_{x}=10^{-5}$.

**Figure 5:**
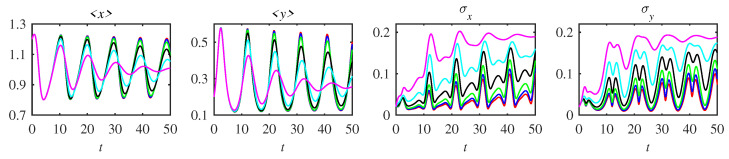
Φ/d=1.2: Scan over $Q_{x}=3\times 10^{-6},10^{-5},3\times 10^{-5},10^{-4},3\times 10^{-4},10^{-3}$ for $Q_{y}=10^{-5}$.

**Figure 6:**
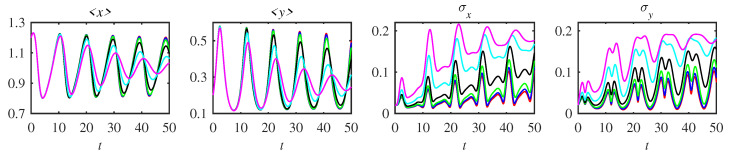
Φ/d=1.2: Scan over $Q_{y}=3\times 10^{-6},10^{-5},3\times 10^{-5},10^{-4},3\times 10^{-4},10^{-3}$ for $Q_{x}=10^{-5}$.

**Figure A1:**
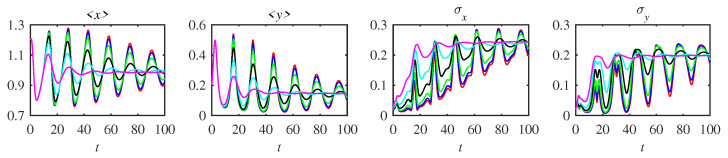
Φ/d=0.8: Scan over $Q_{x}=3\times 10^{-6},10^{-5},3\times 10^{-5},10^{-4},3\times 10^{-4},10^{-3}$ for $Q_{y}=10^{-5}$.

**Figure A2:**
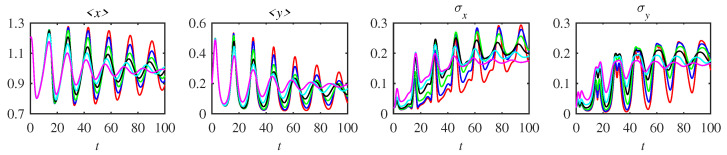
Φ/d=0.8: Scan over $Q_{y}=3\times 10^{-6},10^{-5},3\times 10^{-5},10^{-4},3\times 10^{-4},10^{-3}$ for $Q_{x}=10^{-5}$.

**Figure A3:**
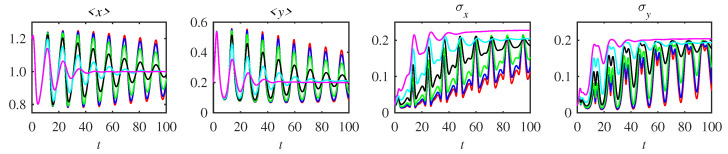
Φ/d=1.0: Scan over $Q_{x}=3\times 10^{-6},10^{-5},3\times 10^{-5},10^{-4},3\times 10^{-4},10^{-3}$ for $Q_{y}=10^{-5}$.

**Figure A4:**
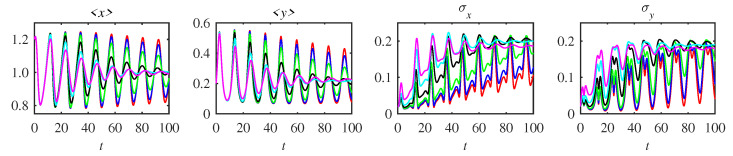
Φ/d=1.0: Scan over $Q_{y}=3\times 10^{-6},10^{-5},3\times 10^{-5},10^{-4},3\times 10^{-4},10^{-3}$ for $Q_{x}=10^{-5}$.

For non-zero stochastic noises, we show time traces of 〈x〉, 〈y〉, $\sigma_{x}$, and $\sigma_{y}$, for the smallest and largest values of Φ/d=0.6,1.2 in Figure 3, Figure 4, Figure 5 and Figure 6 here, while those for Φ/d=0.8, 1.0 in Appendix A (Figure A1, Figure A2, Figure A3, Figure A4). Each plot contains results for different values of $Q_{x}\;\left[Q_{y}\right]=3\times 10^{-6}$ in red, $10^{-5}$ in blue, $3\times 10^{-5}$ in green, $10^{-4}$ in black, $3\times 10^{-4}$ in sky-blue, and $10^{-3}$ in magenta, for fixed $Q_{y}\;\left[Q_{x}\right]=10^{-5}$.

Overall, for all values of $Q_{x},Q_{y}$, the increase in the standard deviation over time is observed due to phase-mixing, while the amplitude of quasi-periodic oscillations gradually decreases over time. This oscillation quenching occurs more rapidly for larger values of $Q_{x}$ or $Q_{y}$, and is more pronounced in the evolution of 〈y〉 than that of 〈x〉, probably because the instability (of *y*) is more susceptible to stochastic noises. Further, compared with $Q_{y}$, $Q_{x}$ is more effective at quenching oscillation amplitude.

For Φ/d=0.6, in comparison with Figure 1, the uncertainty induced by the stochastic noise ($Q_{x},Q_{y}\neq 0$) inhibits *y* from becoming smaller than a certain value depending on $Q_{x}$ and $Q_{y}$. As a result, oscillations become less explosive, as can be seen in Figure 3 and Figure 4. On the other hand, the period of oscillation tends to become smaller for larger values of $Q_{x}$ or $Q_{y}$. A close comparison of the cases with varying $Q_{x}$ and $Q_{y}$ for Φ/d=0.6 reveals that such shortening of the period is more pronounced for increasing $Q_{y}$ for fixed $Q_{x}$ (Figure 4) compared with the case of increasing $Q_{x}$ for fixed $Q_{y}$ (Figure 3).

A similar trend persists for Φ/d=0.8 (see Figure A1 and Figure A2), although the period of oscillation is shortened to a lesser degree in comparison to the case of Φ/d=0.6. This, together with the observation made above, indicates that $Q_{x}$ induces a more severe amplitude quenching while $Q_{y}$ is more effective at shortening the oscillation period, respectively.

In contrast, for Φ/d=1.2 in Figure 5 and Figure 6, $Q_{x}$ and $Q_{y}$ now act in such a way to increase the period rather than decrease it. (A similar tendency can be seen for Φ/d=1.0 in Figure A3 and Figure A4.) This is due to the main difference in oscillation characteristics for Φ/d=0.6 and 1.2. Specifically, for Φ/d=0.6, the oscillations occur less frequently and are explosive, and stochastic noise can turn such rare, explosive oscillations (large ELMs) into more frequent small events (small ELMs). On the other hand, for Φ/d=1.2, oscillations are more regular, being more similar to linear oscillations where a stochastic noise can reduce the oscillation frequency through damping.

### 5.3. Power Loss

**Figure 7:**
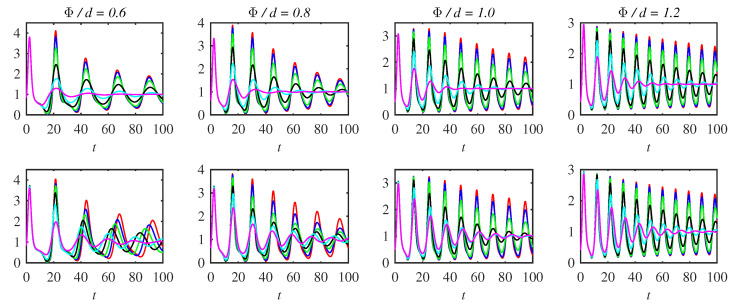
Normalised power loss ${\bar{P}}_{L}$ for Φ/d=0.6,0.8,1.0,1.2 from left to right: Scan over $Q_{x}\;\left[Q_{y}\right]=3\times 10^{-6}$, $10^{-5},3\times 10^{-5},10^{-4},3\times 10^{-4},10^{-3}$ for $Q_{y}\;\left[Q_{x}\right]=10^{-5}$ in the upper [lower] row.

We observed that in the long time limit, $P_{L}\to \Phi$ in all cases. Thus, the normalised power loss ${\bar{P}}_{L}=P_{L}/\Phi$ in Equation (32) gives us a useful measure for comparing different cases of Φ. The normalised power loss ${\bar{P}}_{L}$ is shown in Figure 7 for different cases. The upper [lower] panel shows the results for different values of $Q_{x}\;\left[Q_{y}\right]=3\times 10^{-6}$ in red, $10^{-5}$ in blue, $3\times 10^{-5}$ in green, $10^{-4}$ in black, $3\times 10^{-4}$ in sky-blue, and $10^{-3}$ in magenta for $Q_{y}\;\left[Q_{x}\right]=10^{-5}$, respectively, so using the same colour scheme as before.

Overall, the smaller Φ/d is, the larger the excursion of the normalised power from unity, with larger maxima and smaller minima, similar to what was observed in the deterministic case in Figure 2. This manifests a more explosive nature of the oscillations for smaller Φ. Compared with the deterministic case, the stochastic noise reduces the peak power loss, ${\bar{P}}_{L}^{m}=max\left({\bar{P}}_{L}\right)$, yielding a smaller ${\bar{P}}_{L}^{m}$ for larger $Q_{x}$ and $Q_{y}$. Other notable main effects of stochastic noise $Q_{x}$ and $Q_{y}$ seen in Figure 2 and Figure 7 are as follows. First, as time increases, the peak value and oscillation amplitude of ${\bar{P}}_{L}$ decrease, similar to what was observed in the mean values (Figure 3, Figure 4, Figure 5 and Figure 6). Second, for a given Φ, the peak value ${\bar{P}}_{L}$ monotonically decreases as either $Q_{x}$ or $Q_{y}$ increases. A more significant reduction in the maximum power loss is observed as $Q_{x}$ increases for a fixed $Q_{y}$ (the upper row in Figure 7), compared with the other case of increasing $Q_{y}$ for fixed $Q_{x}$ (the lower row in Figure 7). For instance, for Φ/d=0.6, the values of the second peaks (just beyond t=20) in power loss in Figure 7 are

(33) $$\begin{array}{ccc} & & {\bar{P}}_{L}^{m}=\left(4.11,3.82,3.28,2.46,1.77,1.30\right)\;\mathrm{as}\;Q_{x}\;\mathrm{varies},\\ & & {\bar{P}}_{L}^{m}=\left(4.04,3.82,3.72,3.38,2.81,1.98\right)\;\mathrm{as}\;Q_{y}\;\mathrm{varies}. \end{array}$$

Third, the effects of stochastic noise, noted above, tend to be more pronounced for smaller Φ. This means that the stochastic noises tend to be very effective in quickly suppressing the large power loss associated with large ELMs (Φ/d=0.6). Finally, $Q_{x}$ and $Q_{y}$ shorten [lengthen] the period of the $P_{L}$ oscillations in the case of small Φ/d=0.6,0.8 [large Φ/d=1.0,1.2].

Fourth, the time averages of the normalised ${\bar{P}}_{L}^{m}$ take values ranging from 1.05 for Φ/d=0.6 to 1.02 for Φ/d=1.2, with some differences at the level of a few per cent. This suggests that the input power Φ is a reasonable estimate of the time average of $P_{L}$. However, as noted above, very large fluctuations in $P_{L}$ about the average values (see Figure 7) occur for more explosive cases (Φ/d=0.6), leading to increased power loss due to ELMs.

To demonstrate how $Q_{x}$ and $Q_{y}$ reduce the energy loss $W_{ELM}$ caused by such ELMs, we approximate $W_{ELM}$ by the time-integral of the power loss that is larger than the input power Φ as

(34) $$W_{ELM}/\Phi ∼\left(\Delta t\right)\left({\bar{P}}_{L}^{m}-1\right)/2,$$

where Δt is the duration of the ELM, approximated by the distance between the times where ${\bar{P}}_{L}=1$. The factor of 1/2 accounts for the fact that the peak has a roughly triangular shape going up and down, so estimating $W_{ELM}/\Phi$ as the area of the rectangle $\left(\Delta t\right)\left({\bar{P}}_{L}^{m}-1\right)$ would clearly be an over-estimate.

For instance, we again consider the second oscillation peaks in power loss for the case Φ/d=0.6 in Figure 7, and extract that:

(35) $$\begin{array}{ccc} & & \Delta t=\left(5.65,5.93,6.59,8.03,9.66,11.31\right)\;\mathrm{as}\;Q_{x}\;\mathrm{varies},\\ & & \Delta t=\left(5.72,5.93,5.96,6.10,6.51,7.53\right)\;\mathrm{as}\;Q_{y}\;\mathrm{varies}. \end{array}$$

Using Equations (33) and (35) in Equation (34) then yields:

(36) $$\begin{array}{ccc} & & W_{ELM}/\Phi =\left(8.8,8.4,7.5,5.9,3.7,1.7\right)\;\mathrm{as}\;Q_{x}\;\mathrm{varies},\\ & & W_{ELM}/\Phi =\left(8.7,8.4,8.1,7.3,5.9,3.9\right)\;\mathrm{as}\;Q_{y}\;\mathrm{varies}. \end{array}$$

**Figure 8:**
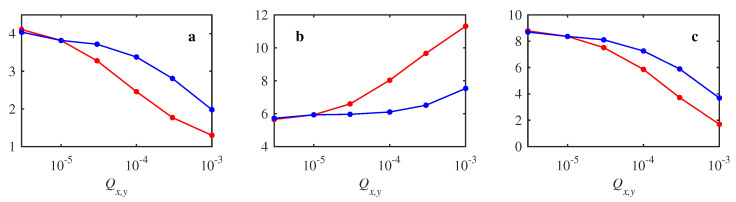
Φ/d=0.6: Panel (**a**) shows ${\bar{P}}_{L}^{m}$ from Equation (33), panel (**b**) shows Δt from Equation (35), and panel (**c**) shows $W_{ELM}/\Phi$ from Equation (36). The horizontal axis is $Q_{x}$ [$Q_{y}$] for the red [blue] curves, corresponding to $Q_{x}$ [$Q_{y}$] varying while the other noise is kept fixed at $10^{-5}$.

Figure 8 shows these quantities in Equations (33), (35) and (36), with red [blue] corresponding to $Q_{x}$ [$Q_{y}$] being the noise that is varying. Both ${\bar{P}}_{L}^{m}$ and $W_{ELM}/\Phi$ in panels (a) and (c) are seen to decrease as $Q_{x}$ and $Q_{y}$ increase, with a greater reduction due to $Q_{x}$ compared to $Q_{y}$. In comparison, Δt has the opposite tendency, its value increasing with $Q_{x}$ and $Q_{y}$, with a more dominant effect of $Q_{y}$.

Finally, we check on the experimental observation that the power loss due to ELMs remains a constant fraction of the input power, $W_{ELM}\cdot f_{ELM}=\left\{0.3-0.4\right\}\times \Phi$, where $f_{ELM}$ is the frequency of ELMs [18,19]. To this end, we focus on the red curves in Figure 7, having $Q_{x}=3\times 10^{-6},Q_{y}=10^{-5}$ in the top row and $Q_{x}=10^{-5},Q_{y}=3\times 10^{-6}$ in the bottom row. We use the second oscillations in each case to extract ${\bar{P}}_{L}^{m}$ and Δt, which then allows us to calculate $W_{ELM}/\Phi$. The next few oscillations give us the period $T_{ELM}$, so then $f_{ELM}=1/T_{ELM}$ gives us the final ingredient to compute the desired quantity $\left(W_{ELM}/\Phi \right)f_{ELM}$.

The first four rows in Table 1 use data from the top row of Figure 7, so $Q_{x}=3\times 10^{-6}$, $Q_{y}=10^{-5}$. The next four rows use data from the bottom row of Figure 7, so $Q_{x}=10^{-5},Q_{y}=3\times 10^{-6}$. The final four rows use data from Figure 2, so the deterministic system with $Q_{x}=Q_{y}=0$. We can see that even quite small amounts of noise reduce the power loss, especially at the smaller Φ/d values where we obtain giant ELMs. For larger noise levels, the reduction in $W_{ELM}$ and, thus, also the power loss becomes even greater, as seen previously in Figure 8.

However, despite such dependence of $W_{ELM}/\Phi$ on $Q_{x}$, $Q_{y}$, and Φ, the power loss due to ELMs $\left(W_{ELM}/\Phi \right)f_{ELM}$ in the last column in Table 1 is within the range of 0.33–0.39, well consistent with the experimental observation 0.3–0.4 [18,19] noted above. In contrast, for a deterministic system (shown in Figure 2), this is no longer the case since $\left(W_{ELM}/\Phi \right)f_{ELM}=0.33$–0.75, taking the value 0.75 for the large ELM Φ/d=0.6. These results thus suggest that the presence of stochastic noise is essential for reproducing the experimental results.

### 5.4. Information Rate

**Figure 9:**
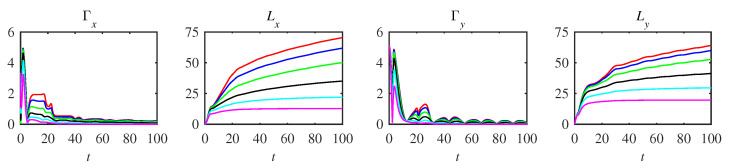
Φ/d=0.6: Scan over $Q_{x}=3\times 10^{-6},10^{-5},3\times 10^{-5},10^{-4},3\times 10^{-4},10^{-3}$ for $Q_{y}=10^{-5}$.

**Figure 10:**
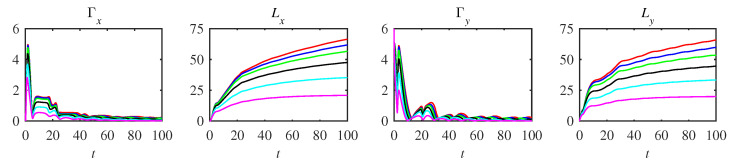
Φ/d=0.6: Scan over $Q_{y}=3\times 10^{-6},10^{-5},3\times 10^{-5},10^{-4},3\times 10^{-4},10^{-3}$ for $Q_{x}=10^{-5}$.

**Figure 11:**
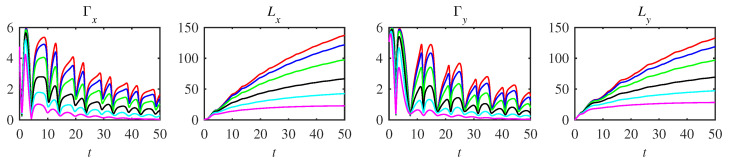
Φ/d=1.2: Scan over $Q_{x}=3\times 10^{-6},10^{-5},3\times 10^{-5},10^{-4},3\times 10^{-4},10^{-3}$ for $Q_{y}=10^{-5}$.

**Figure 12:**
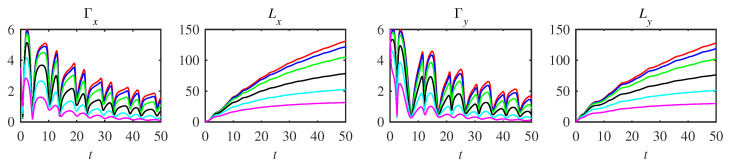
Φ/d=1.2: Scan over $Q_{y}=3\times 10^{-6},10^{-5},3\times 10^{-5},10^{-4},3\times 10^{-4},10^{-3}$ for $Q_{x}=10^{-5}$.

**Figure A5:**
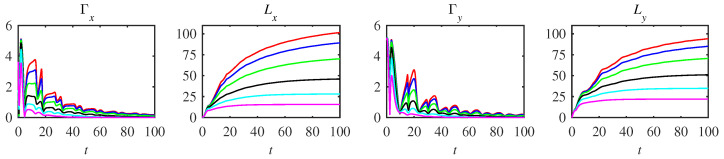
Φ/d=0.8: Scan over $Q_{x}=3\times 10^{-6},10^{-5},3\times 10^{-5},10^{-4},3\times 10^{-4},10^{-3}$ for $Q_{y}=10^{-5}$.

**Figure A8:**
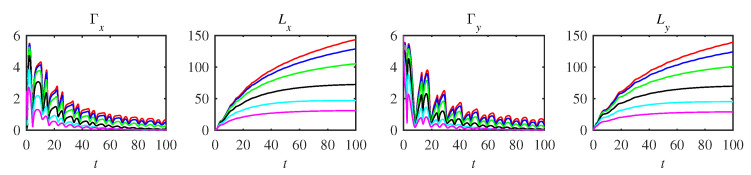
Φ/d=1.0: Scan over $Q_{y}=3\times 10^{-6},10^{-5},3\times 10^{-5},10^{-4},3\times 10^{-4},10^{-3}$ for $Q_{x}=10^{-5}$.

We now examine how the different types of oscillations are reflected in the information geometry, in particular, how the correlation between *x* and *y* inherent in oscillations is captured. Figure 9 and Figure 10 show the time traces of $\Gamma_{x}$, $L_{x}$, $\Gamma_{y}$, and $L_{y}$ for different cases of parameter scanning for Φ/d=0.6, while Figure 11 and Figure 12 are for Φ/d=1.2 (See Figure A5–Figure A8 for Φ/d=0.8,1.0.) In all cases, it is clearly seen that the larger $Q_{x}$ or $Q_{y}$, the smaller $\Gamma_{x}$, $\Gamma_{y}$, $L_{x}$, and $L_{y}$. This is because larger stochastic noise makes the number of statistically distinguishable states smaller. Comparing the upper and lower panels in all cases, $Q_{x}$ seems to cause more decrease in $L_{x}$ and $L_{y}$ compared with $Q_{y}$ due to $Q_{x}$’s stronger oscillation quenching.

**Figure 13:**
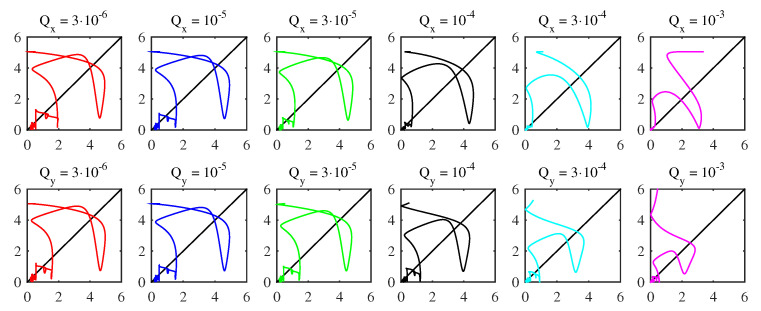
Φ/d=0.6: $\Gamma_{x}$ on the *x*-axis versus $\Gamma_{y}$ on the *y*-axis. The top row shows scans of $Q_{x}$ as indicated at fixed $Q_{y}=10^{-5}$; the bottom row shows scans of $Q_{y}$ as indicated at fixed $Q_{x}=10^{-5}$.

**Figure 14:**
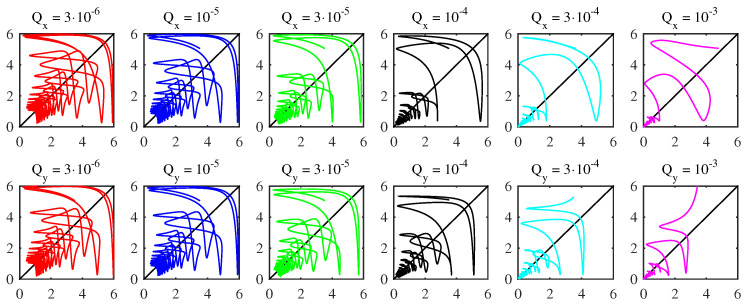
Φ/d=1.2: $\Gamma_{x}$ on the *x*-axis versus $\Gamma_{y}$ on the *y*-axis. The top row shows scans of $Q_{x}$ as indicated at fixed $Q_{y}=10^{-5}$; the bottom row shows scans of $Q_{y}$ as indicated at fixed $Q_{x}=10^{-5}$.

**Figure A9:**
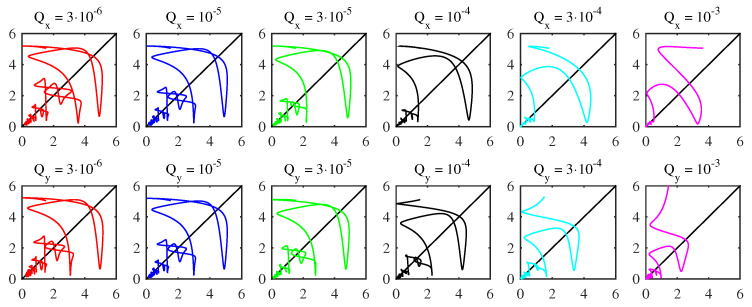
Φ/d=0.8: $\Gamma_{x}$ on the *x*-axis versus $\Gamma_{y}$ on the *y*-axis. The top row shows scans of $Q_{x}$ as indicated at fixed $Q_{y}=10^{-5}$; the bottom row shows scans of $Q_{y}$ as indicated at fixed $Q_{x}=10^{-5}$.

**Figure A10:**
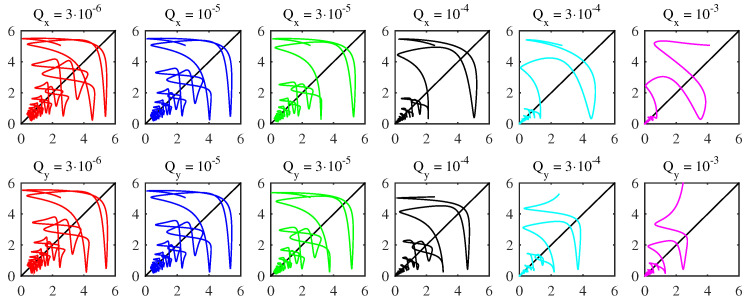
Φ/d=1.0: $\Gamma_{x}$ on the *x*-axis versus $\Gamma_{y}$ on the *y*-axis. The top row shows scans of $Q_{x}$ as indicated at fixed $Q_{y}=10^{-5}$; the bottom row shows scans of $Q_{y}$ as indicated at fixed $Q_{x}=10^{-5}$.

Although the fine features of $\Gamma_{x}$ and $\Gamma_{y}$ are a bit different (e.g., with different oscillation periods), the overall evolution of $L_{x}$ and $L_{y}$ is remarkably similar, suggesting that *x* and *y* undergo similar changes in statistical states despite their different evolutions. To further quantify this correlation, Figure 13 and Figure 14 plot the information phase-portrait, which plots $\Gamma_{y}$ against $\Gamma_{x}$. The diagonal line $\Gamma_{x}=\Gamma_{y}$ is overplotted as the black solid line. The oscillation around $\Gamma_{x}=\Gamma_{y}$ reveals a regulatory interaction between *x* and *y* as a result of overshooting (due to inertial) and restoring forces (due to interaction). Recall that when $\Gamma_{x}$ and $\Gamma_{y}$ cross each other, the time scales of *x* and *y* match with a perfect balance. Such regulatory behaviour is most prominent in the case of Φ/d=1.2 in Figure 14; the large deviation from $\Gamma_{x}=\Gamma_{y}$ in Figure 13 is due to explosive oscillation (intermittency) as well as initial transients. Finally, the larger $Q_{x}$ and $Q_{y}$ are, the less prominent the crossing between $\Gamma_{x}$ and $\Gamma_{y}$ due to loss of phase information (See Figure A9 and Figure A10 for Φ/d=0.8,1.0.)

### 5.5. Entropy Production

**Figure 15:**
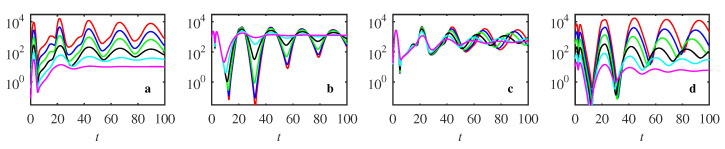
Φ/d=0.6: Panels (**a** and **b**) show ${\dot{S}}_{Tx}$ and ${\dot{S}}_{Ty}$, respectively, as functions of time when scanning over $Q_{x}$ at fixed $Q_{y}=10^{-5}$. Panels (**c** and **d**) show ${\dot{S}}_{Tx}$ and ${\dot{S}}_{Ty}$, respectively, as functions of time when scanning over $Q_{y}$ at fixed $Q_{x}=10^{-5}$.

**Figure 16:**
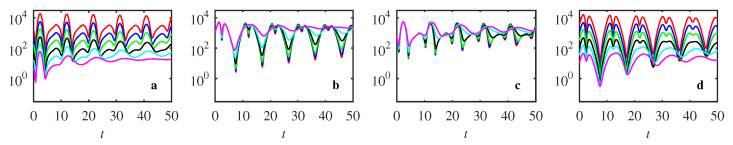
Φ/d=1.2: Panels (**a** and **b**) show ${\dot{S}}_{Tx}$ and ${\dot{S}}_{Ty}$, respectively, as functions of time when scanning over $Q_{x}$ at fixed $Q_{y}=10^{-5}$. Panels (**c** and **d**) show ${\dot{S}}_{Tx}$ and ${\dot{S}}_{Ty}$, respectively, as functions of time when scanning over $Q_{y}$ at fixed $Q_{x}=10^{-5}$.

In both Figure 15 and Figure 16, Panels **a** and **b** show ${\dot{S}}_{Tx}$ and ${\dot{S}}_{Ty}$, respectively, as functions of time when scanning over $Q_{x}$ at fixed $Q_{y}=10^{-5}$; Panels **c** and **d** show ${\dot{S}}_{Tx}$ and ${\dot{S}}_{Ty}$, respectively, as functions of time when scanning over $Q_{y}$ at fixed $Q_{x}=10^{-5}$. What is prominent is that ${\dot{S}}_{Tx}$ and ${\dot{S}}_{Ty}$ in Panels **a** and **d** decrease as $Q_{x},Q_{y}$ are increased, whereas ${\dot{S}}_{Ty}$ and ${\dot{S}}_{Tx}$ in Panels **b** and **c** do not show this tendency. That is, for a smaller value of $Q_{x}$, ${\dot{S}}_{Tx}$ becomes larger while ${\dot{S}}_{Tx}$ changes much less, and vice versus. Mathematically, this appears because ${\dot{S}}_{Tx}$ has $Q_{x}$ in its denominator while ${\dot{S}}_{Ty}$ has $Q_{y}$ in its denominator; $Q_{x}{\dot{S}}_{Tx}$ would be less subject to change when $Q_{x}$ changes. (If *x* were an independent Gaussian variable, $\Gamma_{x}^{2}=Q_{x}{\dot{S}}_{Tx}/\sigma_{x}^{2}+{\dot{S}}_{x}^{2}$). Further, it is useful to note that this different behaviour of ${\dot{S}}_{Tx}$ [${\dot{S}}_{Ty}$] upon the change in $Q_{x}$ [$Q_{y}$] sharply contrasts with the similar behaviour of $\Gamma_{x}$ and $\Gamma_{y}$ in Figure 13 and Figure 14. These results appear to be another manifestation that the geometric measures, such as $\Gamma_{x}$ and $\Gamma_{y}$, better capture the correlations between *x* and *y*, as discussed in Section 5.4 above.

### 5.6. Comparison among Power Loss, Information Rate, and Entropy Production

**Figure 17:**
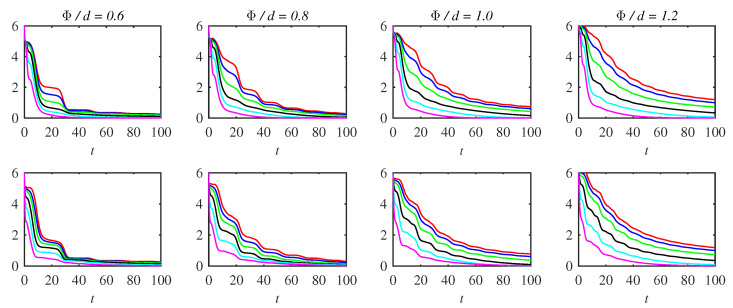
Information rate Γ for Φ/d=0.6,0.8,1.0,1.2 from left to right: Scan over $Q_{x}\;\left[Q_{y}\right]=3\times 10^{-6},10^{-5},3\times 10^{-5},10^{-4},3\times 10^{-4},10^{-3}$ for $Q_{y}\;\left[Q_{x}\right]=10^{-5}$ in the upper [lower] row.

**Figure 18:**
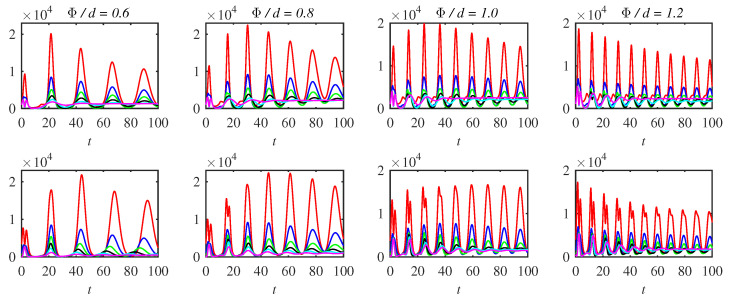
The total entropy production rate ${\dot{S}}_{T}$ for Φ/d=0.6,0.8,1.0,1.2 from left to right: Scan over $Q_{x}\;\left[Q_{y}\right]=3\times 10^{-6},10^{-5},3\times 10^{-5},10^{-4},3\times 10^{-4},10^{-3}$ for $Q_{y}\;\left[Q_{x}\right]=10^{-5}$ in the upper [lower] row.

We considered the individual information rates $\Gamma_{x}$ and $\Gamma_{y}$ calculated from the marginal PDFs of *x* and *y* in Section 5.4 and the individual components of entropy production rate $S_{Tx}$ and $S_{Ty}$ in Section 5.5. One key difference between these measures was noted that $\Gamma_{x}$ is reduced somewhat similarly as either $Q_{x}$ or $Q_{y}$ increases, while $S_{Tx}$ is more severely reduced by $Q_{x}$ than $Q_{y}$. In this section, we examine the statistical measure of the total system by calculating the information rate Γ from joint PDFs (see Equation (Figure 17)) and the total entropy production rates ${\dot{S}}_{T}={\dot{S}}_{Tx}+{\dot{S}}_{Ty}$ (see Equation (23)) and compare them with the power loss in Section 5.3. The results are shown in Figure 17 and Figure 18, respectively, for those cases corresponding to Figure 7.

Notably, Figure 17 exhibits a stair-case-like evolution in time where each large power loss during one ELM oscillation is associated with a region of an almost constant Γ, the value of Γ suddenly decreases from one oscillation to another. If this constant Γ is to be interpreted as a geodesic [32], which was advocated as a signature of self-organisation, ELMs in the presence of stochastic noises can be viewed to occur by jumping from one self-organised state to another.

On the other hand, the overall behaviour of the total entropy production rate ${\dot{S}}_{T}$ in Figure 18 is seen to be well-correlated with that of the power loss in Figure 7, with the maxima of ${\dot{S}}_{T}$ and ${\bar{P}}_{L}$ occurring at similar times, although in some cases, especially for $Q_{y}<Q_{x}$, ${\dot{S}}_{T}$ shows double peaks in the early evolution.

## 6. Conclusions

We conducted a detailed investigation into the effects of various stochastic noises on the statistical properties of ELMs using a time-dependent PDF method and path-dependent information geometry (information rate, information length). Overall, the amplitude of oscillation is diminished over time through the phase-mixing of different trajectories due to either stochastic particle or magnetic perturbations. However, particle perturbation is more effective in this amplitude diminishment compared with magnetic perturbations. In regards to the effects on oscillation frequency, the stochastic noise acts to increase the frequency of more explosive oscillations (large ELMs), while it decreases the frequency of more sinusoidal regular oscillations (small ELMs). In both cases, magnetic perturbations are more effective at altering the oscillation period.

We provided a detailed study of how power loss is affected in different cases. Most of all, a dramatic decrease in power loss for explosive ELMs (small Φ) was observed as $Q_{x}$ and $Q_{y}$ increase, with a more significant effect of $Q_{x}$. The peak power loss and the energy loss due to ELMs were estimated for different Φ, $Q_{x}$, and $Q_{y}$, revealing the important effect of stochastic noises on reducing such losses. However, despite the reduction in $W_{ELM}/\Phi$ by $Q_{x}$ and $Q_{y}$ and their dependence on Φ, the power loss due to ELMs $\left(W_{ELM}/\Phi \right)f_{ELM}$ (in Table 1) for all cases gives 0.33–0.37, consistent with the experimental observation [18,19]. In contrast, for a deterministic system, a deviation from this relation was found for large ELMs. These highlight the importance of a stochastic ELM model for reproducing the experimental results.

Furthermore, the power loss was shown to be strongly correlated with the total entropy production, indicating that the maximum power loss involves a large, irreversible energy dissipation in non-equilibrium. Despite such a strongly non-equilibrium evolution, each ELM oscillation seems to occur in a state where the information rate Γ (calculated from a join PDF) is almost constant, suggesting a geodesic-like behaviour (see [32] and references therein) over one ELM cycle. Envisioning the latter to be a signature of self-organisation Γ [32], the time evolution of ELMs in the presence of stochastic noises is proposed to consist of a sequence of sudden transitions between the different self-organised states.

Another utility of the information geometry in capturing the ELM dynamics is further discussed. Specifically, a strong coupling between *x* and *y* in more regular oscillation is captured by a similar evolution of $L_{x}$ and $L_{y}$, despite the differences in the details of the time-evolutions of the two variables [33]. Furthermore, the information phase-portrait ($\Gamma_{x}$ versus $\Gamma_{y}$) shows that a strong coupling between *x* and *y* can be measured by the oscillation of $\Gamma_{x}$ and $\Gamma_{y}$ around $\Gamma_{x}=\Gamma_{y}$, suggesting the time competition in statistical space. It was also suggested that the correlation is better measured by these information geometric measures than the entropy production rates ${\dot{S}}_{Tx}$ and ${\dot{S}}_{Ty}$ that are affected differently by stochastic noise.

It will be of interest to further extend our analysis to other nonlinear oscillators, including more than one oscillator, to quantify the correlation among different oscillators or emergent phenomena, such as synchronisation. This will require solving the Fokker–Planck equation in higher dimensions, which will be computationally challenging, or further developing stochastic simulation methods for an accurate calculation of information geometry diagnostics [40]. Finally, it would be interesting to investigate more general ELM models.

## Figures and Tables

**Table 1:** Calculations of the relative power loss $\left(W_{ELM}/\Phi \right)f_{ELM}$, as described in the text.

Φ/d	${\bar{P}}_{L}^{m}$	Δt	$W_{\mathrm{ELM}}/\Phi$	$T_{\mathrm{ELM}}$	$\left(W_{\mathrm{ELM}}/\Phi \right)f_{\mathrm{ELM}}$
0.6	4.11	5.65	8.8	22.8	0.39
0.8	3.89	4.08	5.9	15.6	0.38
1.0	3.27	3.69	4.2	11.6	0.36
1.2	2.88	3.42	3.2	9.6	0.33
0.6	4.04	5.72	8.7	23.7	0.37
0.8	3.82	4.13	5.8	15.6	0.37
1.0	3.25	3.71	4.2	11.6	0.36
1.2	2.87	3.43	3.2	9.6	0.33
0.6	15.71	3.07	22.6	30.3	0.75
0.8	7.10	3.31	10.1	16.7	0.60
1.0	4.15	3.41	5.4	11.8	0.46
1.2	2.87	3.40	3.2	9.6	0.33

**Figure A6:**
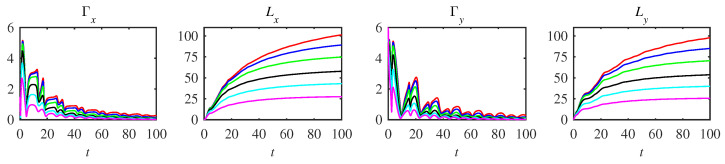
Φ/d=0.8: Scan over $Q_{y}=3\times 10^{-6},10^{-5},3\times 10^{-5},10^{-4},3\times 10^{-4},10^{-3}$ for $Q_{x}=10^{-5}$.

**Figure A7:**
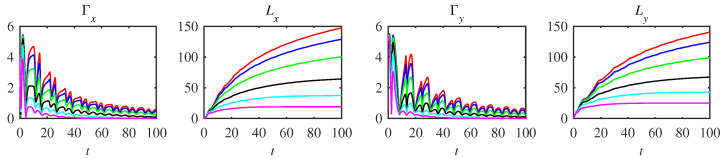
Φ/d=1.0: Scan over $Q_{x}=3\times 10^{-6},10^{-5},3\times 10^{-5},10^{-4},3\times 10^{-4},10^{-3}$ for $Q_{y}=10^{-5}$.

**Figure A11:**
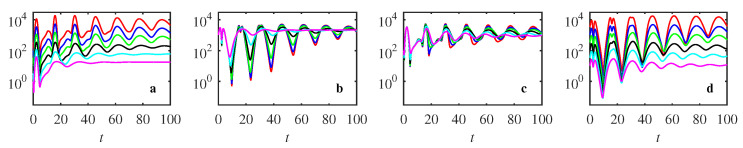
Φ/d=0.8: Panels (**a**,**b**) show ${\dot{S}}_{Tx}$ and ${\dot{S}}_{Ty}$, respectively, as functions of time when scanning over $Q_{x}$ at fixed $Q_{y}=10^{-5}$. Panels (**c**,**d**) show ${\dot{S}}_{Tx}$ and ${\dot{S}}_{Ty}$, respectively, as functions of time when scanning over $Q_{y}$ at fixed $Q_{x}=10^{-5}$.

**Figure A12:**
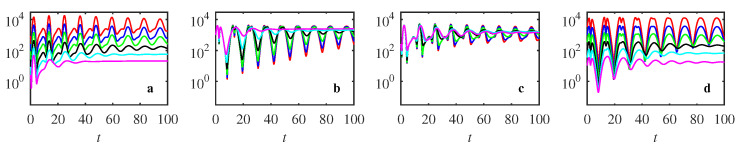
Φ/d=1.0: Panels (**a**,**b**) show ${\dot{S}}_{Tx}$ and ${\dot{S}}_{Ty}$, respectively, as functions of time when scanning over $Q_{x}$ at fixed $Q_{y}=10^{-5}$. Panels (**c**,**d**) show ${\dot{S}}_{Tx}$ and ${\dot{S}}_{Ty}$, respectively, as functions of time when scanning over $Q_{y}$ at fixed $Q_{x}=10^{-5}$.

## Data Availability

The data that support the findings of this study are available from the corresponding author upon reasonable request.

## Appendix A. Complementary Figures

The main text showed results primarily for Φ/d=0.6 and 1.2, the smallest and largest values considered. Results for Φ/d=0.8 and 1.0 may also be of interest though, to show the full range, and how different aspects gradually change. This section, therefore, shows Figure A1–Figure A4 as the equivalents of Figure 3, Figure 4, Figure 5 and Figure 6, then Figure A5–Figure A8 as the equivalents of Figure 9, Figure 10, Figure 11 and Figure 12, then Figure A9 and Figure A10 as the equivalents of Figure 13 and Figure 14, and finally Figure A11 and Figure A12 as the equivalents of Figure 15 and Figure 16.
